# Semi-embedded valve anastomosis a new anti-reflux anastomotic method after proximal gastrectomy for adenocarcinoma of the oesophagogastric junction

**DOI:** 10.1186/s12893-020-00894-6

**Published:** 2020-10-08

**Authors:** Baohua Wang, Yupeng Wu, Haijun Wang, Haiqiang Zhang, Liting Wang, Zhanxue Zhang

**Affiliations:** 1 Thoracic Surgery, The second hospital of Hebei Medical University, Shijiazhuang City, Hebei Province China; 2 Gastrointestinal Surgery, The second hospital of Hebei Medical University, Shijiazhuang City, Hebei Province China

**Keywords:** Adenocarcinoma of the oesophagogastric junction, Proximal gastrectomy, Semi-embedded valve anastomosis, Reflux oesophagitis

## Abstract

**Background:**

There is a high probability of gastroesophageal reflux after laparoscopic proximal gastrectomy for adenocarcinoma of the oesophagogastric junction (AEG). Various anti-reflux anastomotic methods are emerging in clinical practice; however, none of them have been widely accepted. We have innovated a new type of anti-reflux anastomotic method, named semi-embedded valve anastomosis. The aim of this study was to explore the feasibility and anti-reflux effect of the new anastomotic method.

**Methods:**

The clinical data of 28 patients with Siewert II AEG who were treated by semi-embedded valve anastomosis were collected. The key point of the operation is to reconstruct a simulated valve and form an anti-reflux barrier similar to the physiological mechanism. The gastroesophageal reflux disease questionnaire (GerdQ) and classification of gastroesophageal reflux under electron microscopy were used to evaluate gastroesophageal reflux after the operation.

**Results:**

The mean operative duration was 164.3 ± 19.0 min, the median intraoperative haemorrhage volume was 65 ml, the average number of lymph nodes dissected was 23 ± 2.6, the time for valve construction was 15.8 ± 3.2 min, the time for anastomotic reconstruction was 35.4 ± 4.8 min, the median time to first flatus was 3 d, and the median hospitalization duration was 12 d. There was one case of postoperative anastomotic stenosis. The GerdQ score [median (range)] was as follows: 2 (0–6), preoperation; 0 (0–8), 1 month postoperation; 2 (0–12), 3 months postoperation; and 3 (0–12), 6 months postoperation. The Wilcoxon signed-rank sum test was carried out at different times after the operation and the day before the operation, and the differences were not significant. There was one case of grade B gastroesophageal reflux according to the Los Angeles classification system among the gastrofibroscopic re-examination reports of 28 cases.

**Conclusion:**

Semi-embedded valve anastomosis is safe and feasible after proximal gastrectomy for Siewert II AEG and has good anti-reflux effects.

## Background

The incidence of adenocarcinoma of the oesophagogastric junction (AEG) has increased during the past few years in Asia, Europe and the United States [1, 2]. At present, the Siewert method is widely used in AEG typing [3]. The biological behaviour and lymph node metastasis of type I are similar to those of oesophageal cancer, and it is classified as lower oesophageal cancer. Type III is classified as gastric cancer, follows the scope of lymph node dissection of gastric cancer, and is surgically resected by general surgeons through the abdominal oesophageal hiatus. In contrast, type II is traditionally regarded as cardia cancer, because it is located in a special anatomical region; the surgical approach, the scope of lymph node dissection, and the method of reconstructing the digestive tract remain controversial [4, 5].

Whether to perform proximal gastrectomy (PG) or total gastrectomy (TG) is one of the arguments. After PG, the His angle is destroyed because of the removal of the lower oesophageal sphincter, and the anti-reflux barrier is lost; therefore, severe gastroesophageal reflux is likely to occur [6]. In the past, most experts have agreed with laparoscopic TG, but TG can cause nutrition-related complications [7, 8]. Some scholars have proposed various reconstruction methods of anti-reflux anastomosis after PG, such as jejunal interposition [9], double-flap (Kamikawa) anastomosis [10], and tubular gastric anastomosis [11], but most of them have the disadvantages of involving complex fabrication, multiple anastomotic sites, requiring a long time and having a high incidence of potential complications. By using a custom-designed, patented instrument (Chinese patent No. 201220661287.9), we innovated a reverse-puncture method with hand-assisted laparoscopy to perform a new anastomotic method, named semi-embedded valve anastomosis. This anastomotic method is safe, reliable and easy to perform. It forms a one-way valve in principle, which simulates the His angle; therefore, it serves an anti-reflux function.

We conducted a retrospective study to explore the anti-reflux effects of this new anastomotic method.

## Methods

### Patients

A retrospective study of 28 patients with Siewert II AEG who were treated with three united laparoscopic proximal gastrectomies and semi-embedded valve anastomosis in the Department of Gastrointestinal Surgery of the Second Hospital of Hebei Medical University from June 2015 to February 2017 was performed. The inclusion criteria were as follows: Siewert II AEG diagnosed and confirmed by pathological examination; TNM stage I-III; voluntary written informed consent; and treatment with PG combined with semi-embedded valve anastomosis. The exclusion criteria were as follows: extensive tumour infiltration of nearby tissues and organs and distant metastasis; severe or combined endocrine, immune and nervous system disease; treatment with new adjuvant therapy before surgery; and treatment with TG or other anastomotic methods. The staging of cancer was based on the eighth edition of the Union for International Cancer Control. This study was approved by the Ethics Committee of The Second Hospital of Hebei Medical University (2020-R127). Written informed consent was obtained from all patients.

### Three united laparoscopic proximal gastrectomies [12]

**Fig. 1:**
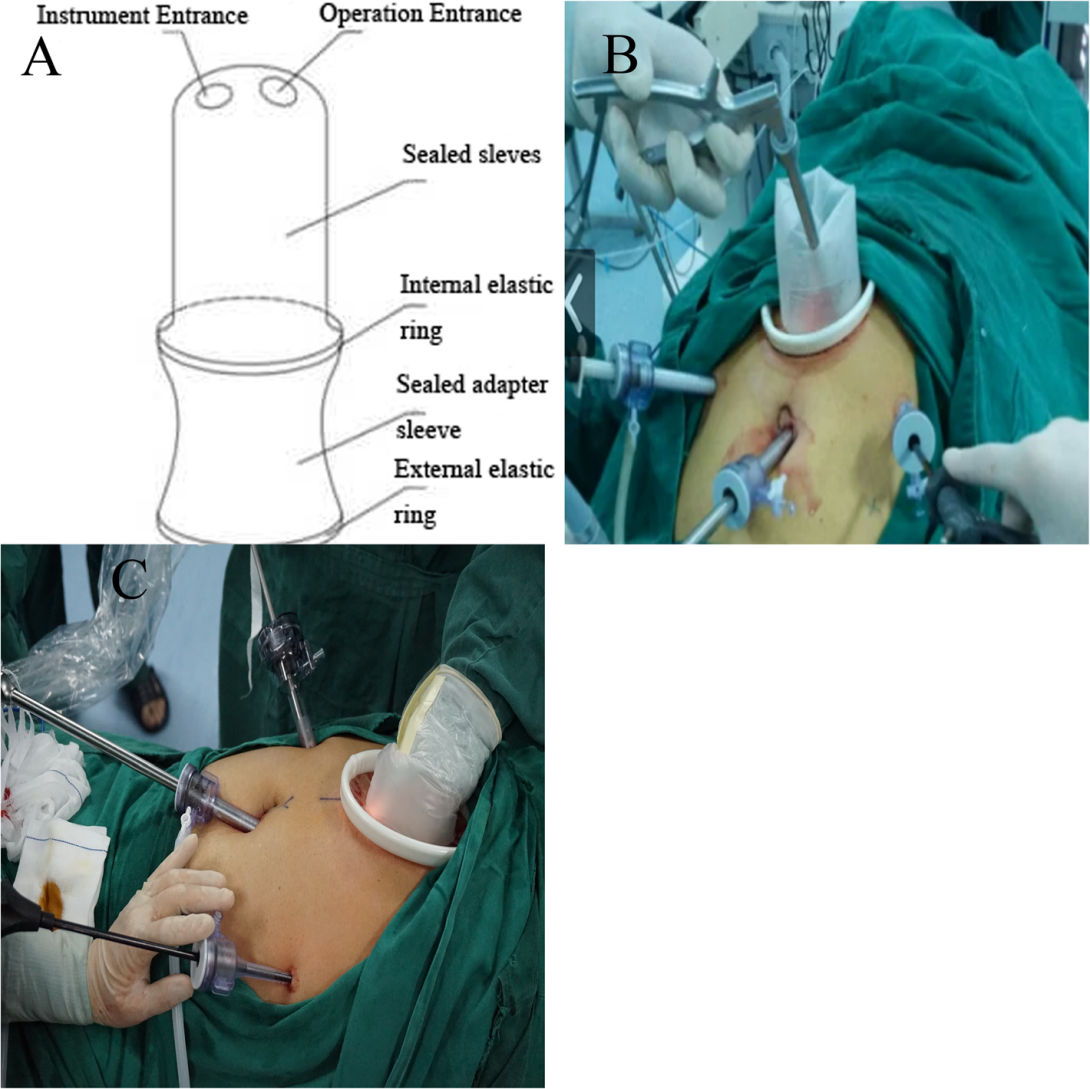
**a** Illustration of our patented device. **b**, **c** From complete laparoscopic to hand-assisted laparoscopic surgery

The stomach was freed with a completely laparoscopic method in advance. With the patient in the supine position, anaesthesia was induced, and routine disinfection and draping were performed. Then, 10-mm, 5-mm and 5-mm trocars were inserted into the lower right abdomen, the lower abdomen and the left abdomen, respectively. The stomach was freed based on *the Japanese Gastric Cancer Treatment Guidelines*. Lymph nodes 1, 2, 3, 4sa, 4sb, 4d, 7, 8a, 9, 11p, 12a, and 20 were dissected. According to the extent of tumour invasion of the oesophagus observed during the operation, the diaphragmatic angle was opened in part of the Siewert II AEG lesion, and the lymph node groups 110, 111 and 112 were dissected through the oesophageal hiatus. The internal elastic ring of the patented instrument was placed in the incision that was 8 cm from the xiphoid process in the middle of the upper abdomen (Fig. 1a), and the external elastic ring was trimmed. Then, the operator placed the left hand in, sealed it with surgical film and fixed it; then, pneumoperitoneum was re-established, and the operator switched to hand-assisted laparoscopy (Fig. 1b, c).

### Principle and key operational points of semi-embedded valve anastomosis

Step 1: The surgeon pinches the lower oesophagus with the assisting hand and transects the left and right vagus nerves.

**Fig. 2:**
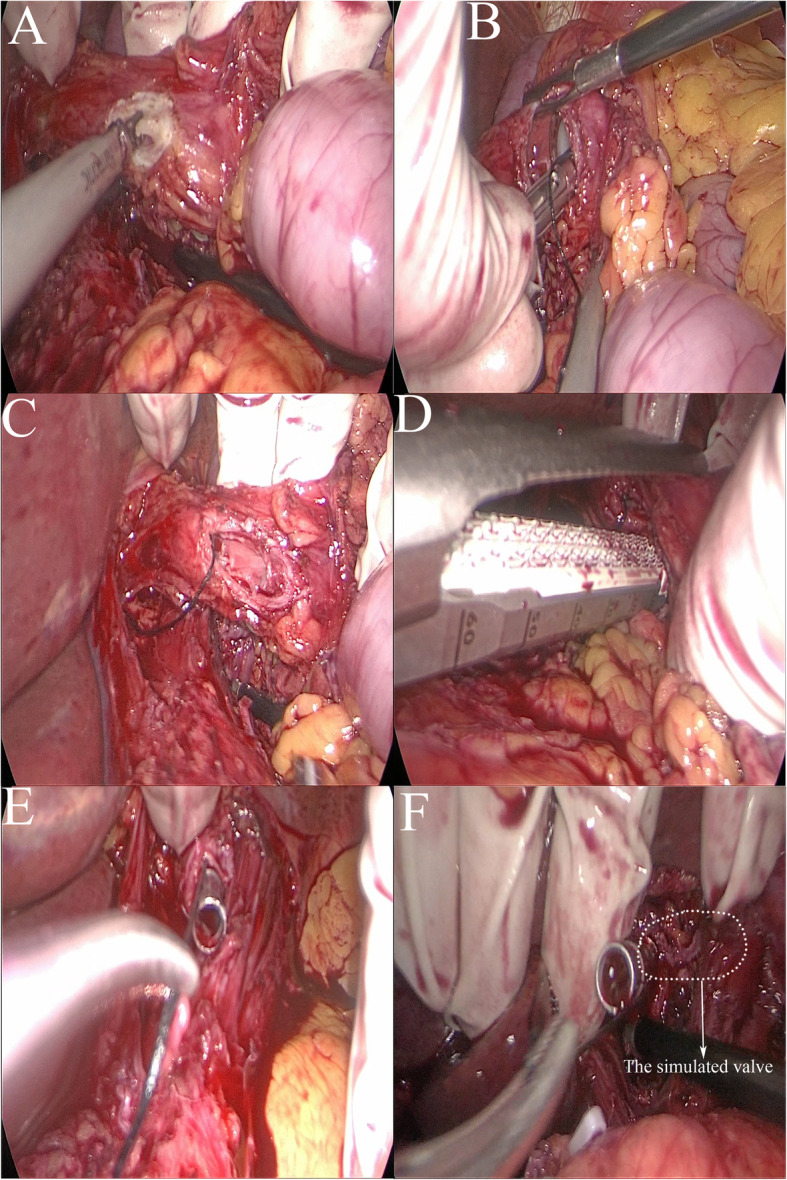
**a** Ultrasonic scalpel opens the anterolateral wall of the oesophagus. **b**, **c** Placement of the nail base under hand-assisted laparoscopy. **d** A 45° oblique cut with a linear stapler. **e:** Reverse puncture. **f** The produced valve (in circle)

Step 2: A hole is opened using an ultrasonic scalpel in the anterolateral wall of the lower oesophagus and then expanded upward and downward (Fig. 2a).

Step 3: The assisting hand is used to hold the edge of the hole to open it with the laparoscopic instrument; meanwhile, the surgeon holds the anastomotic head with the pre-tied string and slowly inserts it into the hole (Fig. 2b, c).

Step 4: Horizontal 45° oblique cut and closure is performed with a linear stapler, with a reverse-puncture opening of 0.3 cm reserved at the lower right corner of the oesophagus (Fig. 2d).

Step 5: The operator uses the laparoscopic instrument or assisting hand to pull the string for reverse puncture (Fig. 2e, f).

Step 6: The patented instrument is removed, the transected stomach is lifted, and the proximal gastric specimen is cut from the greater curvature of the stomach to the lesser curvature of the stomach at approximately 45° to horizontal with a cutter stapler according to the location and size of the tumour.

Step 7: The anterior wall of the remnant stomach is opened under the midpoint of the oblique cut line and anastomosed using a circular anastomat with a reverse-puncture head. During anastomosis, the operator grasps the remnant stomach with the right hand to “push” upward and leftward.

**Fig. 3:**
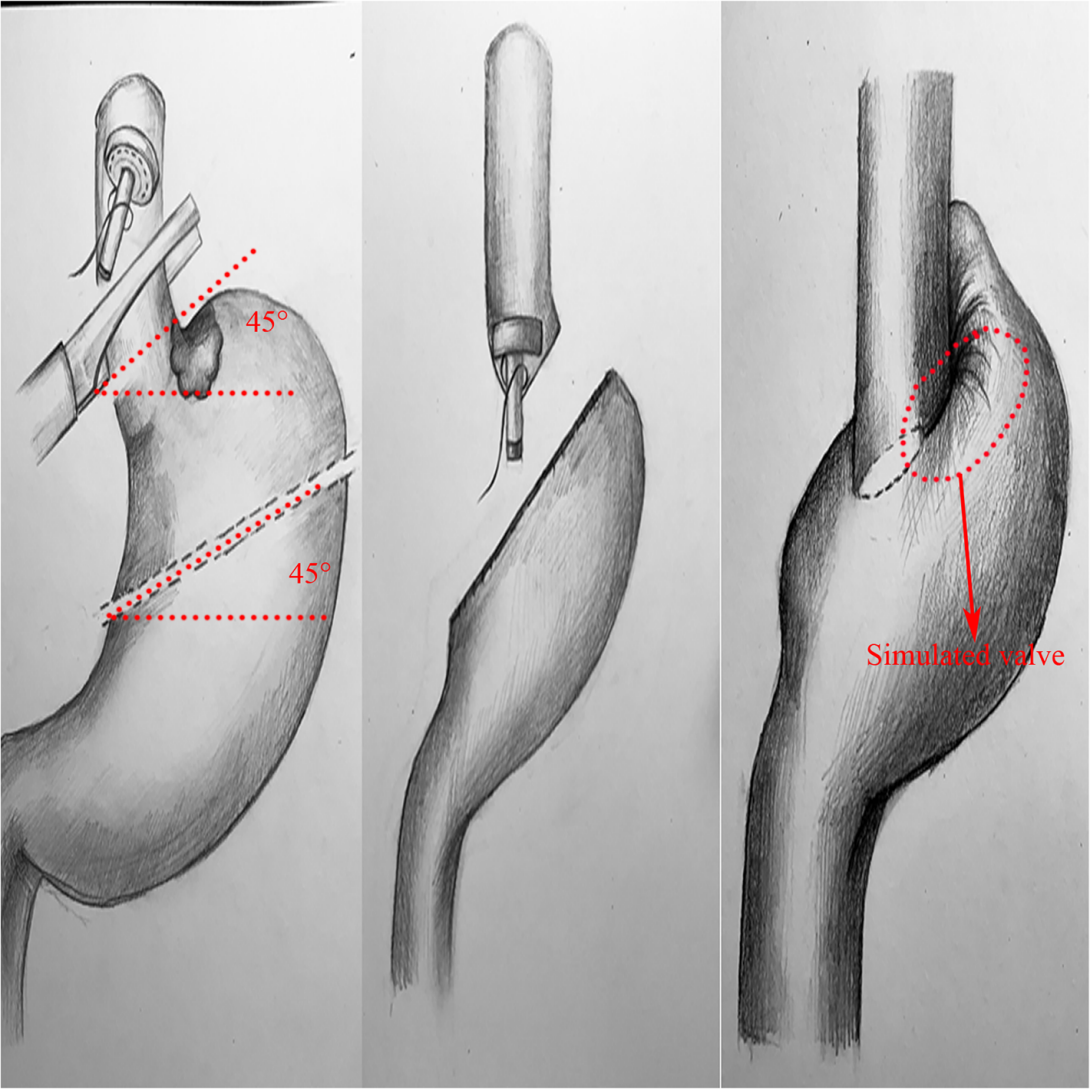
Principle of anastomosis

The principle of anastomosis is shown in Fig. 3.

**Fig. 4:**
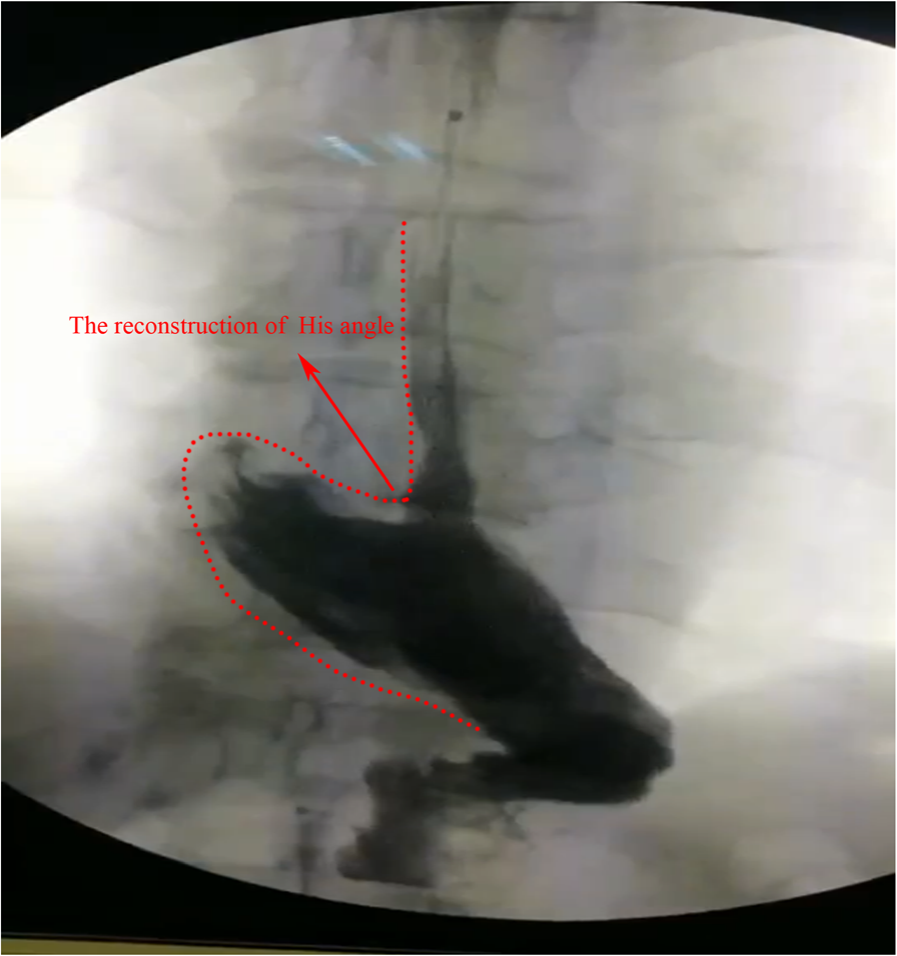
Reconstruction of the gastric fundus and His angle on upper gastrointestinal radiography

The results of upper gastrointestinal angiography in one case after semi-embedded valve anastomosis are shown in Fig. 4. The reconstructed His angle, the reconstructed gastric fundus and the simulated valve structure can be seen by contrast agent filling.

### Data collection

The operative duration, intraoperative blood loss, number of lymph nodes dissected, negative margin rate, time of first flatus, hospitalization duration, and incidence of anastomotic and extra-anastomotic complications were collected from the electronic medical record system and anaesthesia records. The time required for the first anastomosis and the total time required for anastomotic reconstruction were recorded during the operation. The follow-up time was up to now, performed by regular outpatient re-examination and telephone GerdQ gastroesophageal reflux questionnaire, and the classification of gastroesophageal reflux was determined by electron microscopy according to the Los Angeles (LA) classification system for gastroesophageal reflux.

### GerdQ gastroesophageal reflux questionnaire

Symptom score: 0, 1, 2 and 3 points were scored according to “0d”, “1d”, “2–3d”, and “4-7d”, respectively, regarding the frequency of heartburn, reflux, pain in the central upper abdomen and nausea. Influence score: 0, 1, 2 and 3 points were scored according to “0d”, “1d”, “2–3d”, and “4-7d”, respectively, regarding the frequency of sleep affected by gastroesophageal reflux and the frequency of over-the-counter anti-acid drug use by patients. There were six questions in total, and the highest score was 18. GerdQ scores ≥8 indicated a diagnosis of gastroesophageal reflux disease (GERD), and scores ≥3 for the latter two questions indicated the influence of GERD on the quality of life.

## Results

### Patient characteristics and surgical outcomes

The basic data of the patients and the results during and after the operation are shown in Table 1**.** From June 2015 to February 2017, a total of 58 patients underwent surgery for Siewert II AEG; 4 cases of thoracoabdominal surgery, 18 cases of TG, 3 cases of combined multiple organ resection and 5 cases of other anastomotic methods were excluded. Therefore, 28 cases were included. There were 24 males and 4 females, with an average age of 58.9 ± 9.1 (32–74) years and an average BMI of 23.3 ± 2.7. There were 2 cases of gastric cancer stage I, 12 cases of stage II and 14 cases of stage III. Regarding the pathological type, there were 27 cases of adenocarcinoma, including 16 cases of poorly differentiated adenocarcinoma, 5 cases of moderately differentiated adenocarcinoma, 4 cases of highly differentiated adenocarcinoma, and 2 cases of mucinous adenocarcinoma, as well as 1 case of signet ring cell carcinoma. The maximum average diameter of the tumour was 3.3 ± 1.3 cm.

**Table 1:** Patient characteristics and surgical outcomes

Variable	Result
**Age, years, mean ± SD**	58.9 ± 9.1
**Sex, n, male/female**	24/4
**BMI, kg/m**^**2**^**, mean ± SD**	23.3 ± 2.7
**UCII stage, IA/IB/IIA/IIB/IIIA**	1/1/6/6/14
**Diameter of tumour, cm, mean ± SD**	3.3 ± 1.3
**Operative duration, min, mean ± SD**	164.3 ± 19.0
**Estimated blood loss, ml, median (range)**	65 (20–200)
**Number of harvested lymph nodes, n, mean ± SD**	23.0 ± 2.6
**Time to first flatus, days, median (range)**	3 (2–5)
**Postoperative hospital stay, days, median (range)**	12 (9–21)
**Time required for valve construction, min, mean ± SD**	15.8 ± 3.2
**Total time required for anastomotic reconstruction, min, mean ± SD**	35.4 ± 4.8
**Anastomotic complications**	
**Anastomotic leakage**	0
**Anastomotic stenosis**	1
**Other complications**	
**Internal hernia**	1
**Bowel obstruction**	1
**Pancreatic fistula**	0
**Incisional infection**	0
**Peritoneal abscess**	0
**Pneumonia**	1
**Los Angeles grade of gastroesophageal reflux, n, A/B/C/D**	1/1/0/0

Twenty-eight patients were successfully surgically treated with modified radical laparoscopic PG and semi-embedded valve anastomosis. The average operative duration was 164.3 ± 19.0 min, and the intraoperative blood loss was 65 ml. Due to injury to blood vessels around the spleen, one patient underwent combined splenectomy. The average number of lymph nodes dissected was 23.0 ± 2.6. The upper stump tested positive by microscopy in one case, and tested negative in the remaining cases. The time required for valve construction was 15.8 ± 3.2 min, the time required for anastomotic reconstruction was 35.4 ± 4.8 min, the median time to first flatus was 3 d, and the median hospitalization duration was 12 d.

One patient show no abnormalities in receiving a liquid diet after the operation but complained of vomiting after ingesting a large amount of food. Upper gastrointestinal radiography confirmed anastomotic stenosis 18 d after the operation. After endoscopic dilatation treatment, the patient was cured without anastomotic leakage. One 72-year-old patient with chronic obstructive pulmonary disease developed hyperpyrexia and dyspnoea 6 d after the operation, and lung computed tomography confirmed inflammation in bilateral lungs accompanied by arrhythmia and then acute respiratory distress syndrome. The patient was transferred to the intensive care unit for treatment and discharged 1 week later. One patient was admitted to the hospital 1 year after the operation due to strangulated intestinal obstruction caused by internal hernia of the small intestine and was cured after a second operation.

### Indexes of gastroesophageal reflux

The GERD score was successfully evaluated in 28 patients through preoperative conversation, outpatient re-examination and telephone follow-up. The data in each group were matched at different times after the operation with that of the day before the operation. The data in each group did not conform to a normal distribution according to the normality test. Thus, the Wilcoxon signed-rank sum test was used (Table 2). Twenty-eight patients came to the hospital for gastrofibroscopic re-examination half a year after the operation, and according to the LA classification system for gastroesophageal reflux, there was one case of grade A and one case of grade B disease. Patients with reflux oesophagitis regularly took acid-suppressant drugs, such as proton pump inhibitors, and improved after conservative treatment, without and cases of grade C or grade D disease.

**Table 2:** Patient GerdQ scores

Time	preoperation	1 month postoperation	3 months postoperation	6 months postoperation
**GerdQ score, median (range)**	2 (0–6)	0 (0–8)	2 (0–12)	3 (0–12)
**Z**	–	-0.338^a^	-0.851^a^	-1.396^a^
**P**	–	0.736	0.395	0.163

^**a**^**Based on positive rank**

### Postoperative follow-up

Twenty-eight patients were followed up effectively, and the follow-up time was 6–46 months. No patients were lost to follow-up. One patient developed liver metastasis 2 years after the operation, and 3 patients died within 6 months to 2 years after the operation due to tumour recurrence.

## Discussion

Some scholars have advocated TG for Siewert II AEG. Reason 1: Intolerable gastroesophageal reflux has a high probability of occurring with the use of oesophageal gastric-remnant anastomosis after PG. In a study by Nakamura M, the incidence of reflux oesophagitis above grade B in the traditional oesophageal gastric-remnant anastomosis group was 21.8% (12/55) [6], and Chen S reported an incidence of 35.3% (12/34) [13]. Some patients needed to take acid suppressant drugs for a long time, which seriously affected their quality of life. Bile and food repeatedly stimulated the anastomosis and led to the development of anastomotic inflammation, further causing gastric stump cancer. Reason 2: Most AEG tumours are more malignant than other gastric cancer tumours in terms of biological behaviour [14]. To achieve better radical treatment of tumours and reduce recurrence, TG is needed.

Currently, a number of studies have shown that there is no advantage in terms of the postoperative survival rate for Siewert II AEG patients treated with active TG and lymph node dissection. Recently, Yura [15] studied 202 cases and revealed a metastasis rate of 0.99% for lymph node 4d, 0.006% for lymph node 12a, and 0% for lymph nodes 5 and 6 in T2–3 AEG. Kaixuan Zhu [16] et al. analysed the Surveillance, Epidemiology and End Results database, and among 1584 patients treated with PG and 633 patients treated with TG, they found no difference in the 5-year overall survival rate, but PG resulted in better long-term survival in patients over 70 years of age. In this study, most of the patients had disease in a middle stage, including 14 patients with stage IIIA disease; these patients were followed up for 24 months, and 3 died from tumour recurrence. We believe that PG should be cautiously considered in the treatment of patients with stage III disease.

A disadvantage of TG is the possibility of a poor nutritional status, anaemia and dumping syndrome after the operation [17, 18]. Due to the complete removal of gastric parietal cells, the secretion of intrinsic factors can no longer occur, and vitamin B12 absorption is poor, which results in megaloblastic anaemia. With the removal of principal cells, digestive enzymes of the stomach are lost, and nutrition-related complications are further aggravated. A study by Nishigori [19] showed that the weight loss after PG was significantly less than that after TG, and the gastrointestinal symptoms after PG were also less than those after TG. A study by Lee [8] showed significantly better nutritional serological indexes (albumin, total protein, haemoglobin) 2 years after PG than 2 years after TG.

Physiological anti-reflux mechanism: 1. The lower oesophageal sphincter is a high-pressure zone under normal conditions that forms a pressure barrier and blocks reflux. 2. The traction effect of the phrenic oesophageal ligament prevents reflux. 3. The angle formed by the right posterior wall of the gastric fundus and the left side of the abdominal segment of the oesophagus (His angle) is an acute angle; when the pressure in the gastric cavity increases, the expanded gastric fundus compresses the left side of the lower segment of the oesophagus and closes it [20, 21]. PG can destroy the above structure; additionally, due to removal of the vagus nerve, the remnant stomach loses control of the vagus nerve, and gastric peristalsis weakens. The pylorus continues to contract, which inhibits gastric emptying and increases the probability of gastroesophageal reflux.

We were inspired by the mitral valve and tricuspid valve of the heart. When the arterial pressure is high, the blood compresses the valve root to close the opening and prevent the blood from flowing back. Why cannot a valve be made manually at the junction of the oesophageal and remnant stomach by surgery?

In this operation, a 45° oblique cut was first made with a linear stapler; then, the sloped edge of the row of staples formed the initial valve. The reverse-puncture opening was left in the lower right corner. When performing anastomosis with the anastomat rod and the nail base, the nail base was aligned vertically, and the anastomosis was made obliquely on the right side of the simulated valve. The operator grasped the remnant stomach and pushed it to the upper left, which made the deeply embedded the starting section of the valve in the lower left segment of the oesophagus, constructing a new His angle between the left posterior wall of the remnant stomach and the oesophagus. When food is ingested through the mouth, the simulated valve opens. When the pressure in the remnant stomach cavity increases and reflux occurs, the valve root is squeezed on the left side, and the valve closes. The difference in the GerdQ score between before and after the operation was not statistically significant in 28 patients, which preliminarily confirms the anti-reflux effect of the new anastomotic method; however, one patient scored 12 points. After the operation, only one case of reflux oesophagitis above grade B was confirmed by gastrofibroscopy, resulting in a low incidence of 3.57% (1/28).

According to the principle, we divided anti-reflux operational methods after PG into two categories:

1. Method of changing the physiological digestive tract pathway: single-lumen jejunal interposition, pouch jejunal interposition, and double-tract reconstruction.
2. Method of improving the traditional oesophageal gastric-remnant anastomosis: oesophagogastric anterior wall anastomosis, double-flap (Kamikawa) anastomosis, anastomosis of gastroesophageal side overlap, and tubular gastric anastomosis.

Jejunal interposition involves inserting a segment of the pedicled jejunum between the oesophagus and the remnant stomach. The rhythmic peristalsis of the jejunum and the alkaline solution neutralize the residual gastric acid and therefore plays a role in anti-reflux. We think jejunal interposition has some shortcomings. First, the operation is complex, requires a long time to perform, forms three anastomoses, and carries the risk of anastomotic complications. The principle of Kamikawa anastomosis is similar to that of ours, with the aim of forming an embedded valve and constructing a His angle. In 33 cases of Kamikawa anastomosis reported by Kuroda [10], the number of reflux oesophagitis cases above grade B was 0, while the average time required for anastomosis reconstruction was 109 min at the one-year follow-up, without a grade B record. We believe that Kamikawa anastomosis requires cutting of the sarcoplasmic layer of the “H” type, inserting the anastomosis into the submucous layer by hand, and then folding and covering the two layers of the gastric muscle flap. This procedure is also complicated. Excessive overlapping of the valve is likely to cause ischaemic necrosis of the valve or anastomotic stenosis. Yoshito Yamashita [22] et al. reported an anastomotic anti-reflux method of oesophageal-remnant stomach side overlap via a linear cutter stapler under complete laparoscopy. We believe that in this method, a long segment of the lower oesophagus needs to be reserved, which cannot guarantee upper margin R0 resection of Siewert II AEG tumours with dentate line invasion.

There is only one anastomosis in this anastomotic method. The final anastomosis is a circular anastomosis performed under direct vision, which requires less of the reserved lower oesophageal segment and is safer. There were no cases of anastomotic leakage in the 28 treated patients. The principle of our anastomotic method is to construct a one-way valve at the junction of the oesophagus and remnant stomach, which does not affect the circular anastomosis; therefore, we can also combine this method with other anti-reflux surgical methods freely, such as oesophageal and gastric forearm anastomosis and tubular gastric anastomosis, to achieve a “double-guaranteed” anti-reflux effect.

We invented three united laparoscopic surgeries. Using a patented instrument, the operator could freely switch between using single-port laparoscopy, complete laparoscopy and hand-assisted laparoscopy during the operation [12]. In the initial stage, complete laparoscopic surgery was used to free the stomach and dissect the lymph nodes. Then, the operator switched to hand-assisted laparoscopic surgery for anastomosis. The exposure and traction effects of the hand assistance made it more convenient to place the nail base. In this study, the average time required to construct the valve and perform anastomotic reconstruction was only 35.4 min, indicating that this procedure greatly shortened the total operative duration. Sample collection, anastomosis and hand assistance were all performed through the incision made for placement of the patented instrument, making reasonable use of the auxiliary incision.

There are some shortcomings in this method. If the valve is too large, anastomotic stenosis may be caused by the circular anastomat. There may be subjective bias in the GerdQ score of patients in this retrospective analysis, and more objective 24-h pH impedance tests and other experiments are required. Furthermore, there were few cases in this study, which may affect the statistical results.

## Conclusions

Semi-embedded valve anastomosis is safe and feasible after PG for Siewert II AEG, with a short anastomotic time and a certain anti-reflux effect. To further confirm the efficacy of this method, a multicentre, prospective study is needed. In addition, clinicians need to choose the most familiar method of anastomotic reconstruction according to their own conditions.

## Data Availability

The datasets generated and/or analyzed during the current study are not publicly available due to protecting individual patient privacy but are available from the corresponding author on reasonable request.

## Abbreviations

AEG: Adenocarcinoma of the oesophagogastric junction
PG: Proximal gastrectomy
TG: Total gastrectomy
GerdQ: Gastroesophageal reflux disease questionnaire
BMI: Body mass index
GERD: Gastroesophageal reflux disease
LA: Los Angeles
